# Neural cell competition sculpting brain from cradle to grave

**DOI:** 10.1093/nsr/nwaf057

**Published:** 2025-02-20

**Authors:** Yu Zheng Li, Lisen Gao, Xue-Lian Sun, Lihui Duan, Man Jiang, Qing-Feng Wu

**Affiliations:** State Key Laboratory of Molecular Developmental Biology, Institute of Genetics and Developmental Biology, Chinese Academy of Sciences, Beijing 100101, China; State Key Laboratory of Molecular Developmental Biology, Institute of Genetics and Developmental Biology, Chinese Academy of Sciences, Beijing 100101, China; University of Chinese Academy of Sciences, Beijing 100101, China; State Key Laboratory of Molecular Developmental Biology, Institute of Genetics and Developmental Biology, Chinese Academy of Sciences, Beijing 100101, China; University of Chinese Academy of Sciences, Beijing 100101, China; State Key Laboratory of Molecular Developmental Biology, Institute of Genetics and Developmental Biology, Chinese Academy of Sciences, Beijing 100101, China; Department of Physiology, School of Basic Medicine and Tongji Medical College, Huazhong University of Science and Technology, Wuhan 430030, China; State Key Laboratory of Molecular Developmental Biology, Institute of Genetics and Developmental Biology, Chinese Academy of Sciences, Beijing 100101, China; University of Chinese Academy of Sciences, Beijing 100101, China; Beijing Key Laboratory for Genetics of Birth Defects, Beijing Children's Hospital, Beijing 100045, China

**Keywords:** neural cell competition, cellular fitness, neural progenitors, neuronal survival, synaptic competition, glial cell competition, brain development, neurodegenerative diseases, brain tumors

## Abstract

Darwinian selection, operating within the cellular ecosystem of multicellular organisms, drives a pervasive surveillance mechanism of cell–cell competition that shapes tissue architecture and function. While cell competition eliminates suboptimal cells to ensure tissue integrity across various tissues, neuronal competition specifically sculpts neural networks to establish precise circuits for sensory, motor and cognitive functions. However, our understanding of cell competition across diverse neural cell types in both developmental and pathological contexts remains limited. Here, we review recent advances on the phenomenon, and mechanisms and potential functions of neural cell competition (NCC), ranging from neural progenitors, neurons, astrocytes and oligodendrocytes to microglia. Physiological NCC governs cellular survival, proliferation, arborization, organization, function and territorial colonization, whereas dysregulated NCC may cause neurodevelopmental disorders, accelerate aging, exacerbate neurodegenerative diseases and drive brain tumor progression. Future work that leverages cell competition mechanisms may help to improve cognition and curb diseases.

## Teaser

This review explores neural cell competition (NCC), a Darwinian selection process that eliminates suboptimal cells and sculpts neural network, thereby shaping brain development, function and pathology. It covers recent advances in understanding NCC across various neural cell types and its dysregulation in neurodevelopmental disorders, neurodegenerative diseases and brain tumors. Future work leveraging these mechanisms could improve cognition and curb diseases.

Cell competition is a biological process in which cells within a tissue compete for survival and resources. This competition manifests through either the removal of less-fit cells or the increased proliferation and outgrowth of more-fit cells. Cellular fitness indicates the ability of a cell to outcompete its neighbors in terms of survival, proliferation and function [1,2]. Cell competition in both *Drosophila* and mammals takes various forms, including competition for limited spatial resources or trophic factors, mechanical interactions between cells, competition that is mediated by fitness fingerprints and apoptosis activation [3–8]. Cell competition, initially identified and extensively studied in *Drosophila* [9], has been subsequently reported in mouse models. In the developing mouse epiblast, intrinsic heterogeneity in Myc expression levels triggers stem cell competition, in which Myc-enriched cells proliferate, resulting in the apoptotic elimination of less-fit cells [10]. Furthermore, during skin development, competition eliminates less-fit cells through either engulfment by fitter progenitors or expulsion, contributing to the formation of stratified tissue layers [11]. Therefore, cell competition serves as a quality-control mechanism, maintaining proper tissue growth and homeostasis by eliminating compromised cells [12].

## CELL COMPETITION IN THE NERVOUS SYSTEM

Neural cell competition (NCC)—a phenomenon that involves cells within the nervous system that are struggling for survival, resources and space—has emerged as a critical mechanism that shapes brain development and plasticity [13–16]. The earliest conceptualization of neuronal competition dates back to 1980, linking processes such as neuronal death and synaptic elimination, and proposing the trophic factor theory to explain how neurons compete for viability and synaptic connections [17]. Later, the theory of competition for target-derived neurotrophic factors evolved and culminated in 2008 with a model in which two neurotrophin receptors and three neurotrophic factors orchestrate neuronal competition through feedback loops [18–20]. Recent studies on intercellular rivalry between neural progenitors, astrocytes or microglia have demonstrated their competitive interactions through signaling cues, homophilic interactions and phagocytosis mechanisms during development [13,15,16,21,22]. In brief, cell competition in the nervous system spans the entire developmental continuum and entails a cascade of competitive selection events. This starts with neural progenitors that are vying for survival and proliferation, followed by neurons that are striving to survive and establish connections, and culminates in neuroglia that are competing for limited space and resources [13–16]. NCC provides surveillance on the fitness of neural progenitors, neurons and glial cells, and shapes the synaptic efficacy of neural circuits during neurodevelopment [16,23–25]. Specifically, cellular competition between neural progenitors regulates neural tissue patterning and adjusts the stem cell pool, potentially influencing brain morphogenesis and size [13]. Neuronal, synaptic and glial competitions subsequently remodel neural wiring, synaptic connections and the microenvironment, facilitating the formation of precise functional circuits and sculpting the final neural architecture [13,26]. Besides trophic factor theory, structural connection, neuronal activity and intrinsic fitness fingerprints have been proposed to underpin the mechanisms of NCC. The manifestation of cell competition extends beyond cellular viability and quantity, encompassing the robustness of synaptic connections, the complexity of process arborization and the extent of territorial dominance [3,9,27]. A deeper understanding of the molecular mechanisms that underlie NCC will provide valuable insights into enhancing cognitive function, improving mental health and advancing therapeutic strategies for treating neural diseases.

## CELL COMPETITION BETWEEN NEURAL PROGENITORS

**Figure 1.**
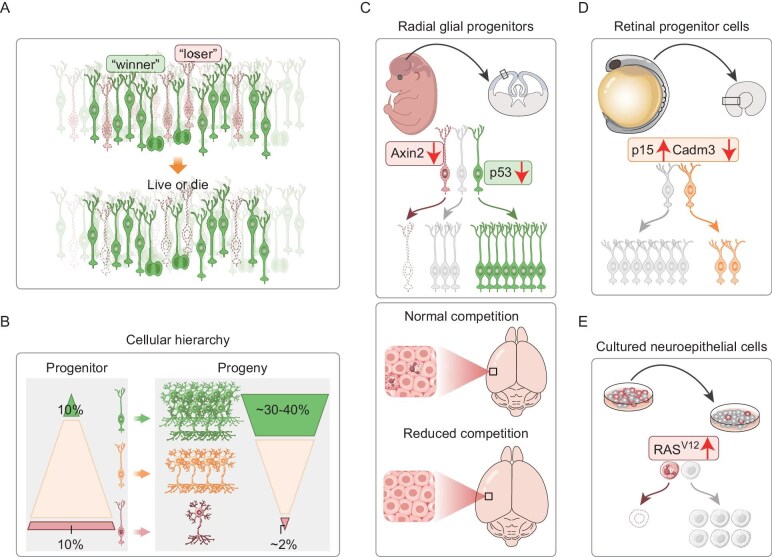
Competition between NSPCs. NSPC competition can manifest in (A) survival of only ‘winners’ or (B) differences in the numbers of progeny. Expression levels of key genetic regulators can influence the outcome of NSPC competition. (C) In the mouse brain, Axin2-mutant NSPCs become losers while p53-deficiency confers winner status [13]. Optimal elimination of ‘losers’ results in normal tissue size development. Conversely, the absence of NSPC competition may result in tissue enlargement. (D) Imbalanced expression of p15 induces competitive interactions among retinal progenitor cells during zebra-fish development. When p15-overexpressing cells are transplanted into wild-type host embryos, they form smaller clones [22]. (E) Overexpression of RAS^V12^ in cultured neuroepithelial cells results in a competitive disadvantage against normal cells, causing apoptosis [21].

Neural stem/progenitor cells (NSPCs) in the developing brain arise from the ventricular zone and undergo a sequential transition from symmetric to asymmetric cell division. Limited space and niche signals likely drive competitive interactions between NSPCs, ensuring proper neuronal generation and circuit assembly. Recently, paired clonal analysis demonstrated that ∼24%–30% of NSPCs in the forebrain were potentially eliminated by their isogenic siblings, indicating endogenous cell competition (Fig. 1A) [13]. Further analysis revealed a cellular ecological hierarchy in which the top 10% of proliferative NSPCs generate 30%–40% of progeny cells, while the bottom 10% generate only 1%–2% (Fig. 1B) [13]. Such cell competition differs from the lateral inhibition in both mechanism and outcome. While cell competition acts as a cell-fitness surveillance mechanism, lateral inhibition is considered a patterning mechanism that regulates binary cell fate choice, yielding a ‘salt and pepper’ distribution pattern of distinct cell types [28,29]. In contrast to Notch-mediated lateral inhibition, which promotes the maintenance of the neural progenitor cell pool [29–31], cell competition leads to the elimination of unfit NSPCs.

Recent studies, through genetic mosaicism *in vivo* or cell-mix co-culture assays *in vitro*, identified several genetic regulators of cell competition, including the Axin2–p53 axis, Cadm3 and juvenility-associated genes [13,21,22]. In the context of genetic mosaics in the forebrain, Axin2-mutant NSPCs become loser cells, undergo p53-dependent apoptosis and can be engulfed by winner cells (Fig. 1C) [13]. Mechanistically, Axin2 interacts with p53 as a scaffold protein and compromises its post-transcriptional stability. Inhibition of the p53 signaling pathway rescues the viability of Axin2-mutant NSPCs, enabling their clonal expansion in the mosaic environment. Coincidentally, p53-deficiency alone in NSPCs confers a winner status, enhancing proliferative potential and inducing the apoptotic elimination of neighboring unfit cells. Winner cells that lack p53 often neighbor dying loser NSPCs, implying that NSPC competition may rely on cell–cell contact. Notably, homogeneous shifts in either Axin2 or p53 levels do not alter the viability of NSPCs, indicating that competition arises from disparities in the expression of these key drivers. Similarly, imbalanced p15 expression triggers competitive interaction between retinal progenitor cells in developing zebra fish [22]. Wild-type donor cells that are transplanted into p15-overexpressing embryos expand clonally, whereas p15-overexpressing cells that are transplanted into wild-type host embryos generate smaller clones (Fig. 1D). However, germ-line overexpression of p15 minimally affects fully differentiated retina formation, suggesting the role of p15 as a cell competition driver. In p15-overexpressing clones, the ectopic introduction of bcl2a halted apoptosis but did not boost progeny cell numbers [22], suggesting that p15-mediated cell competition relies on non-apoptotic cell-death mechanisms, such as ferroptosis or necroptosis. The *in vitro* cell-mix assay is also instrumental in elucidating the mechanisms that underlie cell competition. For example, mixing p53-deficient NSPCs with wild-type cells conferred a growth advantage to NSPCs with p53 deficiency [13]. In contrast, ectopic expression of oncogenic RAS^V12^ in immortalized NSPCs induces a loser state when co-cultured with normal cells, leading to apoptotic cell death *in vitro* (Fig. 1E) [21]. Together, these studies suggest that cell competition arises from preexisting intrinsic differences in expression levels or mutational landscapes of fitness-related genes.

Given the critical role of NSPCs in neural cell generation and brain homeostasis, cell competition between NSPCs is proposed to precisely control cell number generation and brain volume. Despite variable neuronal output from individual NSPCs, proliferative NSPCs develop a competitive mechanism to coordinate division behavior, ultimately contributing to the construction of an invariant brain [32]. Specifically, NSPCs could adjust their division mode and rate based on the proliferative behavior of neighboring cells, potentially through the homophilic interaction of cell adhesion molecules [22]. While it was initially suggested that cell competition does not affect total tissue size, clear evidence demonstrates that the cumulative abrogation of multiple cell competition drivers enhances neuronal production and enlarges brain size [13]. A plethora of cell competition inducers, including those in WNT, NF-κB, Hippo, mTOR and JAK-STAT signaling [33–38], are also implicated in brain size regulation, with genetic variants, mutations or deletions in these pathways linked to macrocephaly or microcephaly [39–44]. However, due to the critical roles of these pathways in governing cell proliferation, survival and metabolism, it remains challenging to disentangle whether germ-line mutations in these inducers contribute to abnormal brain size through impaired cell competition or altered cell growth. Importantly, de novo mosaic mutations in components of the PI3K-Akt-mTOR signaling have been shown to cause megalencephaly or hemimegalencephaly [43,45,46], whereas germ-line mutations in this pathway seem to result in milder changes in brain size [47–49]. Given that genetic mosaicism creates sharper differences between competing cells than germ-line mutations, it may provoke stem cell competition during early neurodevelopment, promote the emergence of super-competitors and drive unchecked expansion of the neural progenitor cell pool, ultimately resulting in megalencephaly. These human genetic studies suggest the potential involvement of cell competition as a mechanism that underlies brain malformation.

From the evolutionary perspective, hominid brain expansion occurs in parallel with changes in craniofacial features, such as flattened face, reduced prognathism and smaller teeth [50]. It is likely attributed to a competitive imbalance between neural crest stem cells and NSPCs, which both originate from a common neuroepithelial source, leading to more neuroepithelium being directed toward brain development at the expense of neural crest formation [50,51]. Moreover, cell competition, which pursues a stereotyped principle of selecting and expanding the most appropriate cells [52], has undoubtedly boosted clonal evolution of NSPCs over millions of years. Regardless of biological conditions or environmental factors, such selective pressure would have favored the emergence of sustainably proliferative NSPCs, thereby driving the evolutionary expansion of brains across species. Such a hypothesis is supported by the role of Dpp/BMP signaling in cell competition, along with the observation that evolutionarily increased BMP7 expression in cortical NSPCs contributes to mammalian cortex expansion [53–55].

Another study demonstrated that a mechanical ‘tug of war’ between neuroepithelial cells propels neural plate folding during neural tube closure, in which apically constricted cells at plate hinges elongate and expand neighboring cells [56]. While this involves only shape change, mechanical force may convey cell status information to neighbors, prompting the senescence and apoptotic elimination of neighboring cells. Coincidently, a line of senescent cells was observed at the closing of a neural tube [57]. These studies collectively suggest that competitive interaction between NSPCs, driven by either genetic imbalance or mechanical force, may ensure proper neural patterning, such as neural tube closure, and optimize brain size.

## CELL COMPETITION BETWEEN NEURONS

As the intensity of cell competition between NSPCs wanes during the transition from the proliferative phase to differentiation [13], a subsequent wave of competition between neurons arises. This phase encompasses not only survival, but also the establishment of dendritic and synaptic connections within neural circuits. During embryonic development, the brain undergoes a period of neuronal overproduction, followed by a selection process in which unfit neurons are eliminated [26]. In humans, the number of cortical neurons peaks during late gestation, followed by a significant reduction to a stable level around birth [58]. Similarly, in the rodent cerebral cortex, an estimated 25%–35% of excitatory pyramidal neurons undergo programmed cell death, while ≤40% of inhibitory interneurons are eliminated during the early postnatal period [59,60].

**Figure 2.**
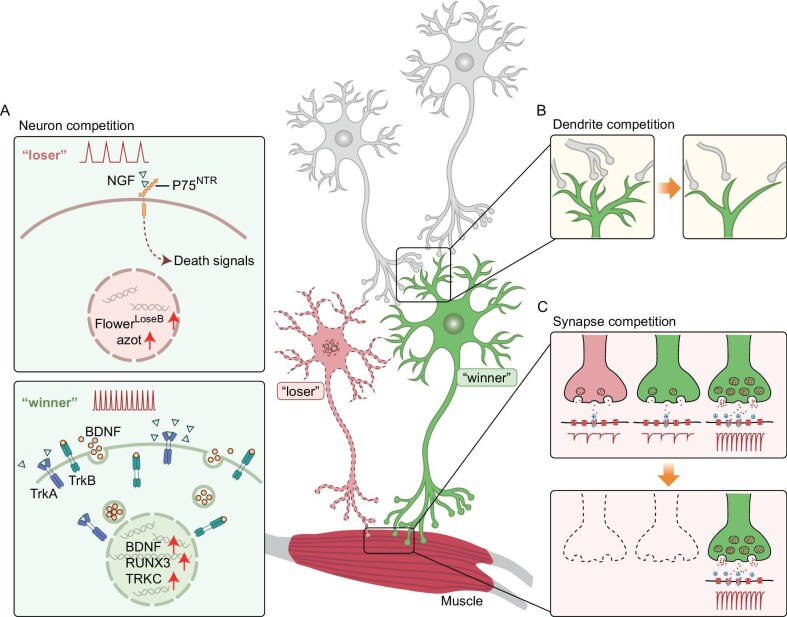
Neuronal competition. Neuron competition involves various mechanisms, including neurotrophic signaling, circuit integration, electrical activity and intrinsic fitness. (A) Neurons compete for survival by securing neurotrophic factors such as NGF. ‘Losers’ undergo apoptosis through NGF-p75 receptor binding while ‘winners’ attract more NGF, triggering retrograde signals that activate survival transcription factors and increase their intrinsic fitness. Electrical activity also stimulates BDNF production and secretion, enhancing neuronal competitiveness. (B) In dendrite competition, excessive dendritic branching initially exists, some of which is eliminated via competition. More connections mean a more successful competitor. Increased glutamate receptor GluD2 and glutamatergic input offer protection from pruning/elimination. Increased RhoA results in losers being pruned. (C) In synapse competition, multiple initially innervated axonal branches compete until only one remains. Established synapses receive more resources, weakening competing branches. Postsynaptic firing strengthens and stabilizes a synapse. JAK2 tyrosine kinase plays a key role in synapse elimination.

Molecular and cellular analyses have identified multiple mechanisms that underlie interneuronal competition, which involve neurotrophic factor signaling, neuronal circuit integration, electrical activity and intrinsic fitness (Fig. 2A). First, the concept of developing neurons competing for limited target-derived neurotrophic factors to ensure survival has been well established [17,61,62]. In sympathetic or dorsal root ganglia, winner neurons that innervate their target tissues receive a greater abundance of trophic signals (e.g. nerve growth factor (NGF)), which bind to their receptors and trigger retrograde signaling to activate survival-promoting transcription factors [20,63]. These neurotrophic factors further modulate competition by promoting the expression of their own receptors in winner neurons—a self-reinforcement mechanism [19]. Additionally, NGF induces the release of death signals through the p75 neurotrophin receptor to eliminate neighboring unfit cells [19], establishing a cross-repression mechanism. Brain-derived neurotrophic factor (BDNF) has also been implicated in mediating competitive interactions among cortical excitatory neurons, vestibular neurons and retinal ganglion cells [61,64,65]. Second, adult-born excitatory neurons in the dentate gyrus of the hippocampus compete for integration into existing circuits. Their survival depends on establishing strong connections with input and output neurons, which are then rewarded with increased neurotrophic support [14]. Third, as electrical activity can stimulate BDNF production and secretion, an updated hypothesis proposes that neuronal activity dynamically regulates survival and death by enhancing immature neuronal circuit integration and trophic factor release [27,61]. Lastly, intrinsic neuronal fitness can also mediate cell competition independently of target-derived trophic signals, adding another layer of complexity [26,66]. Studies in the mouse dorsal root ganglion revealed that nascent sensory neurons exhibit transcriptional heterogeneity prior to apoptotic elimination [26]. Competition selects for neurons with elevated retinoic signaling, RUNX3 activity and tropomyosin receptor kinase C (TRKC) expression, promoting survival and circuit integration. In the *Drosophila* retinal epithelium, a ‘Flower code’ has been identified as a postmitotic cell selection mechanism. The flower gene encodes three cell membrane protein isoforms, with the Flower^LoseB^ isoform specifically tagging loser neurons for elimination [66]. As another loser signature gene, *azot* acts as a ‘cell-fitness checkpoint’ by measuring relative Flower^LoseB^ levels [12]. These findings suggest that the intrinsic fitness of a neuron may be genetically encoded.

In the developing cortex, direct evidence that demonstrates cell competition for survival between excitatory neurons or between inhibitory interneurons remains lacking. Nonetheless, numerous studies have shown that neuronal activity plays a fundamental role in promoting the survival of both excitatory and inhibitory cells [59,67–69]. For interneurons, synaptic connections with excitatory neurons are required for survival [59,70], as extended by recent findings that extrinsic signals from excitatory neurons support the survival of somatostatin but not parvalbumin interneurons [71]. These studies imply that neuronal activity and circuit integration collectively determine the fitness of cortical neurons. However, it remains unclear whether and how more active neurons suppress the activity of nearby, less-active or less-well-connected neurons, leading to selective death. Notably, transplantation experiments that show comparable viability between grafted TrkB-deficient and wild-type interneurons in the host brain argue against intercellular competition for the neurotrophic factor BDNF [60]. Thus, further studies would be required to elucidate the phenomenon and mechanisms that underlie the life-or-death rivalry between cortical neurons.

Immature neurons not only compete for survival, but also strive for dendritic growth and synaptic integration. In the cerebellum, glutamatergic synaptic connections between parallel fibers and Purkinje cells are critical for motor learning and coordination, with the glutamate receptor GluD2 expressed postsynaptically [72]. Sparse knockout of GluD2 specifically in Purkinje cells disrupts the efficacy of synaptic input from parallel fibers and leads to stunted dendritic outgrowth [72]. Interestingly, global knockout of GluD2 does not alter Purkinje cell dendrite morphology [73]. Conversely, mosaic overexpression of GluD2 in a subset of Purkinje cells grants them a competitive advantage, allowing them to exhibit excessive dendritic branching and establish more synaptic connections [73]. As such, beyond interneuronal competition, intraneuronal competition exists between dendrites and synapses for circuit integration during neurodevelopment (Figs 2B and 2C). At early postnatal stages, neurons establish exuberant dendritic arborizations with numerous synaptic connections, which are subsequently refined through a pruning process that is driven by activity-dependent dendritic and synaptic competition, ultimately shaping neural circuits [74–76].

Activity-dependent dendritic competition is driven by the input of various neurotransmitters, including glutamate, GABA and acetylcholine (Fig. 2B). In the mouse olfactory bulb, mitral cells initially project superfluous apical dendrites that converge onto a single glomerulus and then prune in an activity-dependent manner [75,76]. Robust glutamatergic input to a developing dendrite locally inhibits RhoA activity and offers protection from pruning [75]. Subsequent neuronal depolarization triggers a neuron-wide activation of RhoA, leading to the elimination of other apical dendrites in the mitral cell. In *Drosophila*, motoneurons receive excitatory cholinergic inputs and inhibitory GABAergic inputs in the proximal and distal dendritic domains, respectively [77]. Increased expression of postsynaptic cholinergic receptors promotes the growth of proximal dendrites, while overexpression of GABAergic receptors favors distal dendritic length at the expense of proximal regions [77]. Notably, the total dendritic length or branching of the neuron is not altered, suggesting competition for dendritic building material. These studies demonstrate that dendritic competition sculpts neuronal connections by favoring strong inputs and eliminating weaker branches.

Neural networks undergo continuous remodeling through activity-dependent synaptic competition (Fig. 2C), which occurs across various brain regions and persists across the lifespan [78]. Within a single neuron, different synapses compete for stabilization or survival—a process that is regulated by local synaptic activity [78,79]. The concept of activity-dependent synaptic competition arose from studies of ocular dominance segregation in the visual cortex and has since been observed in neuromuscular junctions, barrel cortex, auditory cortex and olfactory bulb [78–81]. In the developing cerebellum, Purkinje cells are initially innervated by multiple climbing fibers, but only a single ‘winner’ climbing fiber is selectively strengthened and maintained, while the other synapses are eliminated [82–84]. Mechanistically, Hebb's rule suggests that repetitive presynaptic activation coupled with postsynaptic firing strengthens and stabilizes a synapse [85], whereas non-coincident or silent activity is hypothesized to weaken and eliminate synapses [86]. In the presynaptic terminals of the cingulate cortex, JAK2 tyrosine kinase was identified as a critical regulator of inactive synapse elimination [87]. An inhibitory signal may be transmitted from active to inactive synapses, triggering JAK2-dependent elimination of the inactive synapse. Postsynaptic molecules (e.g. Ephrin-B3 or BDNF) have been shown to regulate synaptic density in a competitive context through trans-synaptic cell adhesion interactions or neurotrophic signaling [88]. It is worth mentioning that Hebb's rule favors stronger synapses in dominating postsynaptic activation, creating a positive feedback loop that can trigger runaway activity and fuel vicious synaptic competition [89]. To circumvent the limitations of Hebb's rule, heterosynaptic plasticity upregulates the synaptic weights of inactive synapses near active ones over a broad timescale, allowing the retention of at least some inactive synapses [79,90,91]. Moreover, synaptic metaplasticity enables silent synapses to be more sensitive to activation and primed for a rapid increase in synaptic strength, facilitating the formation of new connections [92]. Therefore, Hebbian plasticity, heterosynaptic plasticity and metaplastic mechanisms work together to create a conducive environment for synaptic competition.

Collectively, developing neurons integrate microenvironmental, electrical, transcriptional and subcellular signals to achieve activity- or connectivity-dependent selection, promoting the elimination of weaker competitors while protecting stronger connections for establishing functional neural circuitry.

## CELL COMPETITION BETWEEN NEUROGLIA

**Figure 3.**
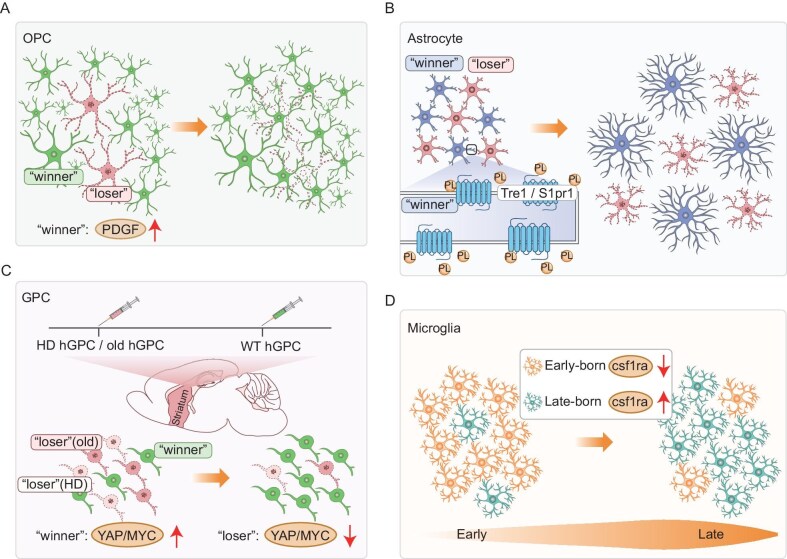
Glial cell competition. (A) In OPC competition, the elimination of ‘loser’ OPCs results in the remaining cells competing for the newly available space. Increased availability of platelet-derived growth factor reduces cell death and promotes population expansion. (B) Astrocytes compete for local phospholipids (PL) mediated by the lipid-binding receptor Tre1/S1pr1. Loss of Tre1/S1pr1 disrupts process elaboration and normal morphogenesis. (C) In GPC competition, WT hGPC outcompete and replace Huntington's disease hGPC and old hGPC, regulated by differences in YAP/MYC expression levels. (D) In microglia competition, age-related dynamic competition exists where the decline in csf1ra expression in early-born microglia over time compromises their competitive fitness compared with late-born microglia.

Glial cells, encompassing astrocytes, oligodendrocytes and microglia represent a substantial portion of the mammalian brain. Within this cellular community and microenvironment, glial cell competition refers to interactions between specific glial subtypes that influence the dominance of certain populations. During brain development, oligodendrocyte progenitor cells (OPCs) arise from three distinct germinal zones in sequential waves [93]. Targeted ablation of a single OPC population results in compensatory responses from the remaining cells without compromising overall development or survival in mice [93], suggesting potential competition for space between OPCs of different origins (Fig. 3A). Other than spatial competition, it has also been implicated that OPCs in the developing rat optic nerve compete for limited amounts of platelet-derived and insulin-like growth factors to survive [94]. It was further hypothesized that these survival signals may originate from axons to promote OPC viability, ensuring that the number of oligodendrocytes matches the axonal lengths that require myelination [95]. OPCs compete not only among themselves, but also with NSPCs from the subventricular zone in demyelination disease models, with both working to repair white matter lesions [96].

Interestingly, astrocytes compete for local phospholipids, mediated by the lipid phosphate phosphatases Wunen/Wunen2 and the lipid-binding receptor Tre1/S1pr1, to promote their process elaboration (Fig. 3B) [15]. This implicates the presence of inter-astrocyte territorial competition. Interspecies chimerism models further support the concept of glial competition. Human glial progenitor cells (hGPCs) that are transplanted into newborn mice extensively engraft and progressively replace the murine glial cells of the host [97], contrasting with the elimination of human pluripotent stem cells by their mouse counterparts in a co-culture assay [98]. In another chimeric mouse model, xenografted healthy hGPCs, with upregulated Yes-associated protein/Myc proto-oncogene (YAP/MYC), outcompete and replace both Huntington's disease-affected and older isogenic wild-type hGPCs *in vivo* (Fig. 3C) [99].

Microglia, unlike astrocytes and oligodendrocytes that are derived from NSPCs, originate from the yolk sac in mammals and infiltrate the brain during early embryogenesis [100,101]. Early microglial progenitors display heterogeneity in their proliferative potential for clonal expansion. They compete for space to occupy specific niches, with a small fraction generating larger clones (Fig. 3D) [102]. Genetic inhibition of microglial apoptosis via BCL2 overexpression significantly increases microglial density while reducing the individual territory that is occupied by each microglia, suggesting that spatial competition shapes microglial colonization [102]. In zebra fish, early- and late-born microglia are generated sequentially from distinct sources [16]. Early-born microglia diminish in adulthood, whereas late-born microglia take over for lifelong maintenance. During this transition, an age-dependent decline in *csf1ra* expression in early-born microglia compromises their competitive fitness when coexisting with late-born microglia [16]. Together, glial cells from diverse spatial and temporal origins, as well as distinct ages and genotypes, may engage in competition for clonal colonization or spatial territories during development, regeneration and disease. Elucidating the mechanisms underlying glial competition holds promise for advancements in regenerative medicine and potential rejuvenation strategies for aging brains.

## ABERRANT COMPETITION CONTRIBUTES TO NEURODEVELOPMENTAL DISORDERS

The impact of disrupted NCC on neurodevelopmental diseases is not well studied. At the cellular level, de novo mosaic mutations in the developing brain may trigger cell competition and drive the emergence of super-competitors among NSPCs [43,45,46]. As aforementioned, these super-competitors could undergo unchecked clonal expansion, potentially resulting in megalencephaly or hemimegalencephaly. Analyses of young post-mortem autistic brains have demonstrated an abnormal excess of neurons in the prefrontal cortex [103], implying a disruption in the cell competition mechanism during autism development. Somatic mosaic mutations in neurons within a relevant lineage may disturb competition with neighboring normal neurons for synaptic innervation, potentially contributing to neurodevelopmental disorders such as epilepsy and schizophrenia [104]. Nevertheless, this hypothesis still requires experimental validation in future studies.

On the synaptic scale, neuroligins, which are cell adhesion molecules that are implicated in autism, have been found to mediate synaptic competition. Consistently with the competition hypothesis, mosaic deletion of neuroligin-1 in cortical neurons reduces their synaptic density, whereas global loss of neuroligin-1 has no effect on synapse numbers [105]. In a mouse model of autism with neuroligin-3 deletion, synaptic competition between climbing fiber inputs on Purkinje cell dendrites is disrupted, leading to ectopic climbing fiber synapses [106]. More importantly, synaptic competition promotes coordinated spine pruning and maturation, with defects in this process associated with developmental neurological diseases such as autism [107,108]. Together, these studies provide valuable insights into the role of NCC in neurodevelopmental diseases at both cellular and synaptic levels.

## CELL COMPETITION DECAYS DURING AGING AND STRAYS IN NEURODEGENERATIVE DISEASES

**Figure 4.**
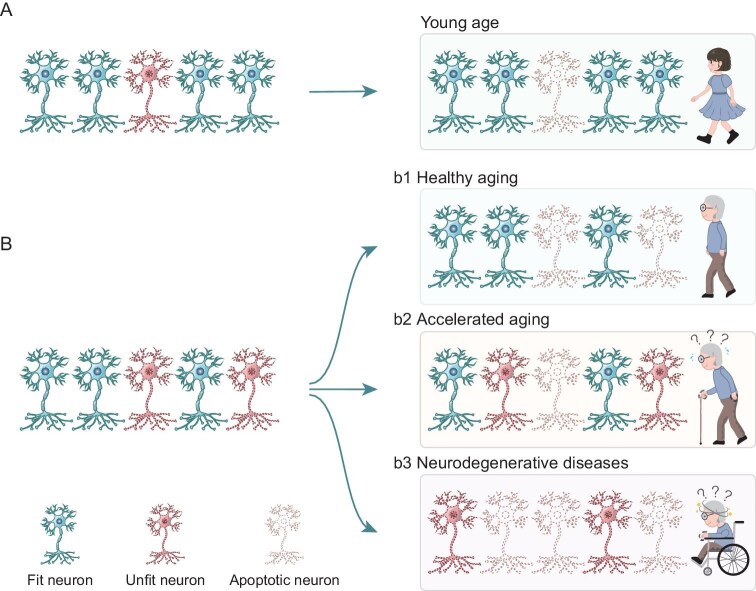
Cell competition during aging and in neurodegenerative diseases. (A) Efficient elimination of unfit neurons by cell competition occurs in young individuals. (B) Additional unfit neurons appear due to aging. (b1) In healthy aging, fit neurons survive whereas unfit neurons are eliminated via apoptosis; this results in better memory strength and precision. Increased neuronal activity and better connectivity increase cell survival and motor/cognitive function. (b2) Impaired cell competition can lead to accelerated aging by allowing unfit neurons to accumulate due to inefficient elimination. (b3) Neurodegenerative diseases are often characterized by neuronal loss that results from the accumulation and apoptosis of unfit neurons—a process that is frequently exacerbated by declining cell competition mechanisms. Age-related oxidative stress, protein misfolding and specific fitness fingerprints (e.g. Flower^LoseB^ and azot) may influence the onset of neurodegenerative diseases.

While unfit neurons are effectively eliminated during the prepubertal stage, the progressive decline in cellular fitness during aging exacerbates imbalances within tissues and organs, potentially triggering cell competition as a quality-control mechanism (Fig. 4A and **4**B). Asynchronous cellular senescence, in which individual cells within a niche age at different rates, may be a key driver [109]. In the mammalian epidermis, premature aging disrupts protein stability, causing differential expression of COL17A1 among epidermal stem cells, which promotes the expansion of clones with high fitness (i.e. high COL17A1) and the elimination of less-fit neighbors [110]. Similarly, lifelong cell selection occurs in *Drosophila*, with unfit cells marked for apoptosis via azot signaling [12]. Disruption of azot function leads to the accumulation of unfit cells and neurodegeneration, which accelerates aging, whereas enhancement of azot activity promotes loser-cell elimination, delays aging and extends the lifespan [12]. Within the nervous system, it has recently been proposed that cell competition contributes to healthy aging by selectively eliminating unfit cells [111,112], contradicting the traditional view that neuronal loss impairs brain health (Fig. 4B) [113]. However, direct evidence for neuronal competition in the aging brain remains elusive. Despite a limited understanding of how neurons assess their fitness and trigger loser-cell elimination, competition dynamics could be harnessed to improve memory precision during aging [14]. In the dentate gyrus, artificial manipulation and transient reduction in the synaptic activity of aged granule neurons weakens their competition with adult-born neurons. This allows the survival of more adult-born neurons, rejuvenating the aged hippocampus and ultimately enhancing memory strength and precision [14].

Neurodegenerative disorders, including Alzheimer's, Parkinson's and Huntington's diseases, are characterized by the accumulation of unfit neurons and progressive neuronal loss (Fig. 4B). The outcome of cell competition between neurons may be influenced by the differential susceptibility of distinct neuronal subtypes to age-related oxidative stress and protein misfolding aggregates [3,111], potentially impacting neuronal survival and degeneration. In the entorhinal cortex, neurons demonstrate heightened vulnerability to disruptions in electrical activity, leading to axonal degeneration that is influenced by competitive interactions between active and inactive neurons [114]. In *Drosophila* models of neurodegenerative diseases, ectopic expression of amyloid-β42 and HuntingtinQ128 recapitulates aspects of Alzheimer's and Huntington's diseases, respectively, leading to the emergence of unfit neurons and the upregulation of Flower^LoseB^ expression [115,116]. Over time, these unfit neurons in the *Drosophila* eye imaginal discs undergo apoptotic cell death in both cell-autonomous and non-cell-autonomous manners. Notably, suppression of the relative differences in Flower^LoseB^ levels between cells significantly reduces the apoptotic elimination of neurons that are expressing aberrant proteins. This finding supports the hypothesis that cell competition, mediated by fitness fingerprints (e.g. Flower^LoseB^ expression), contributes to the removal of unfit neurons. Consistently with the potential benefits of azot-dependent cell competition during aging, the introduction of an additional functional copy of azot in the fly model promotes the apoptotic elimination of unfit neurons and improves motor and cognitive function [12,116]. These studies suggest that NCC contributes to maintaining the integrity of the aged brain and slowing the progression of neurodegenerative processes by selectively eliminating unfit neurons. A deeper understanding is crucial for developing therapeutic strategies that modulate NCC pathways, promote cellular fitness and preserve tissue homeostasis.

## EMERGENCE OF SUPER-COMPETITORS IN BRAIN TUMORS

**Figure 5.**
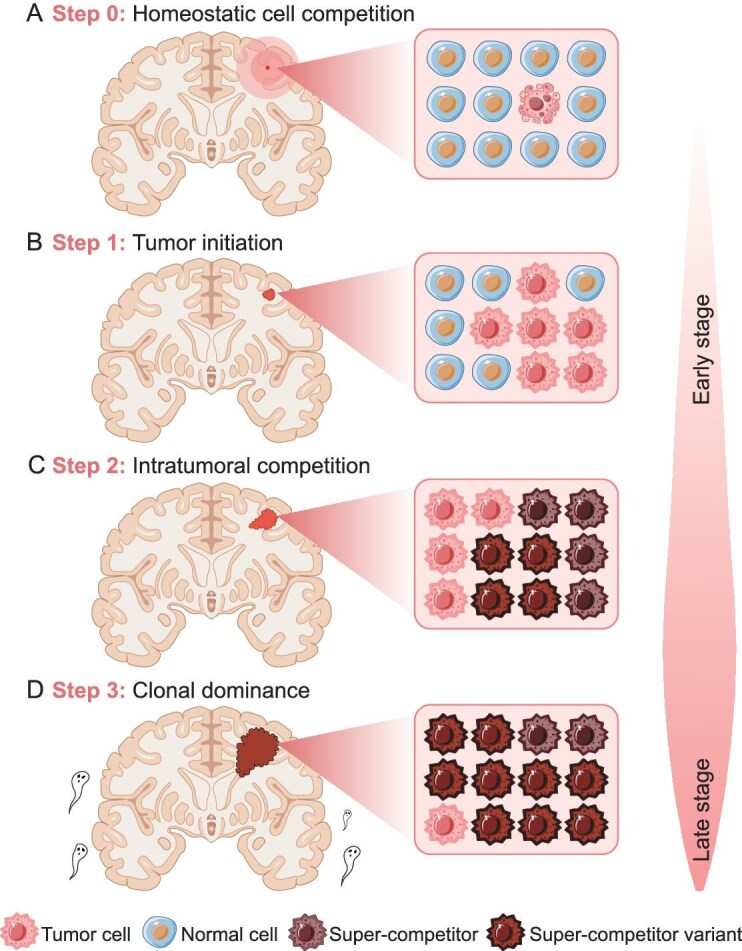
Cell competition between glioma cells. (A) Normal cell competition can detect and eliminate aberrant cells to maintain tissue homeostasis and prevent tumorigenesis. (B) Tumor-initiating cells can reshape their microenvironment to maintain their super-competitor status over normal cells and induce tumorigenesis. (C) Glioma development leads to subclonal diversity, triggering competition between different subclones for limited resources, where super-competitors may have elevated YAP levels, thereby enhancing growth and promoting neighbor apoptosis. (D) In the tumor ecosystem with intense selective pressure, more competitive and aggressive clones gradually become dominant.

Surveillance and clearance of abnormal or transformed cells are essential for maintaining tissue homeostasis and preventing tumor initiation (Fig. 5A) [117]. Cell competition has been proposed as a safeguard against tumorigenesis due to its role in detecting and eliminating aberrant cells [118]. Normally, cells in epithelial tissues sense and actively eliminate newly transformed cells through extrusion, engulfment or apoptotic elimination—a process known as epithelial defense against cancer [119,120]. However, transformed cells can transition into super-competitors and undergo clonal expansion at the expense of normal cells, when they harbor oncogenic mutations that confer a selective fitness advantage [121–123]. In this context, homeostatic cell competition is disrupted, fostering a microenvironment that is conducive to tumor initiation. In the tumor ecosystem, with its intense selective pressure, cancer cells may even hijack cell competition mechanisms to drive clonal competition and evolution, allowing the dominance of more competitive and aggressive clones [124,125]. The quality-control mechanisms in the brain have remained largely unknown. As a suppressor of Wnt signaling, mutations in Axin2 have been shown to promote the development and progression of colorectal cancer [126]. In contrast, the loss of Axin2 in NSPCs leads to competitive apoptosis. This suggests that Axin2-mediated neural stem cell competition may serve as a surveillance mechanism, helping to prevent the occurrence of brain tumors [13].

Glioma is the most prevalent neoplasm in the central nervous system across all ages, accounting for ∼30% of all brain tumors and an overwhelming 80% of malignant brain tumors [117,127]. The cellular origin of gliomas remains controversial, but emerging evidence indicates that NSPCs are particularly susceptible to a series of oncogenic mutations, suggesting them as potential cells of origin for glioma [128]. In cell-mix co-culture assays, adult NSPCs with oncogenic alterations were found to outcompete their wild-type counterparts and even induce quiescence in neighboring normal cells through cell–cell contact and Notch signaling [21,22]. Thus, the tumor-initiating cells could remodel their microenvironment to sustain their super-competitor status (Fig. 5B). As glioma progresses, intratumoral heterogeneity among subclones arises and drives clonal competition for limited space and nutrients (Fig. 5C and 5D) [129,130]. For example, heterogeneous expression of YAP in glioblastoma induces cell competition, leading to the elimination of YAP^Low^ tumor cells and the clonal dominance of YAP^High^ cells [131]. Unbiased clonal tracing with genetic barcoding in glioblastoma models has further corroborated clonal-based cell–cell competition, showing early extensive clonal extinction and the progressive dominance by a small number of clones [132]. Transcriptional analysis identified Myc and its transcriptional targets as critical molecular signatures for clonal dominance.

Medulloblastoma—the most common malignant pediatric brain tumor—originates in the cerebellum or medulla and exhibits tremendous biological heterogeneity [133]. Primary, recurrent and metastatic medulloblastomas have distinct genetic makeups, likely shaped by clonal competition and selection [134–136]. Single-cell genomic and spatial transcriptomic profiling has revealed intratumor clonal complexity and clone-specific cell behaviors, including differences in proliferation, differentiation and immune infiltration [134,137]. While targeted chemotherapy exerts selective pressure, a preexisting minor clone may outcompete previously dominant, rapidly proliferating clones, survive clonal selection and ultimately emerge as the new dominant clone in medulloblastoma relapse [135]. Mechanistically, selective pressure drives transposon insertions in drug-resistant clones, disrupting key tumor suppressors and fostering clonal dominance. Identification of clonal transposon insertions in metastatic medulloblastoma also supports the model in which metastasis arises from a minor subclone of the primary tumor [136]. Thus, clonal evolution that involves transposon insertions contributes to intratumor heterogeneity, promoting clonal competition and selection in medulloblastoma.

Collectively, competitive interactions between transformed cells and microenvironmental cells, as well as between tumor subclones, may regulate the tempo of tumor initiation, progression, recurrence and metastasis.

## UNRESOLVED QUESTIONS AND FUTURE PERSPECTIVES

While trophic factor theory has long been proposed to elucidate neuronal competition, our understanding of when, where, how and why cell competition occurs in the central nervous system under physiological and pathological conditions is still in its infancy. Regarding cellular competition between NSPCs, there are many unresolved questions. For example, what are the diffusible ligands and surface receptors that mediate crosstalk between winners and losers? Is the status of winners and losers dictated by intrinsic fitness genes or environmental factors? What determines whether the loser NSPCs are eliminated or enter a quiescent state? To what extent does transposon-mediated somatic mosaicism regulate NSPC competition and the clonal dominance of winner cells? Does stem cell competition drive the evolutionary expansion of the mammalian brain? Does NSPC competition contribute to the anisotropic proliferation of neighboring clones and thereby cortical folding? Several hypotheses have been put forward to explain cell–cell competition mechanisms: the ligand capture model, surface contact model and diffusible signal model [9]. The ligand capture model posits that cells compete for limited survival factor, whereas the surface contact model suggests that cells report their fitness to neighbors via surface proteins [53,138]. In the diffusible signal model, winner cells release death signals to eliminate losers; alternatively, losers may secrete trophic signals to support the proliferation of winner cells [34,37]. Future studies should explore the models that are employed by NSPCs to mediate competitive interactions, as well as the relationship between intrinsic machinery and extrinsic factors. Advancing our understanding of the molecular mechanisms underlying NSPC competition requires systematic single-cell analysis of NSPCs across developmental stages, brain regions and species. The identification of putative molecular signatures of loser cells [13], combined with the emergence of spatial transcriptomics, proteomics and metabolomics at single-cell resolution, will enable the prediction of neighboring NSPC fates during competition. These spatial multi-omics techniques could also identify potential regulators of NSPC competition. Recent advancements in tools, such as transposon-based genetic mosaicism induction, targeted Perturb-seq and mosaic analysis with a double-markers system [136,139,140], will greatly facilitate our efforts in screening and validation. With the advent of 4D cellular physiology, the simultaneous imaging of nascent RNA and cellular fate in brain slices or organoids promises real-time monitoring of how the transcription dynamics of potential regulators drive competitive interaction between NSPCs. Furthermore, the potential role of asynchronous cellular senescence in NSPC competition remains an intriguing question. During embryonic mammalian development, cellular senescence occurs at multiple sites, often within signaling centers that regulate tissue patterning, such as limb-bud formation and neural tube closure [57,141]. This suggests that developmental senescence differs from the process that is observed during aging, as it is a programmed rather than stochastic event. Future work will shed light on whether cellular senescence contributes to NSPC competition in a niche-dependent manner.

For interneuronal competition, it remains challenging to distinguish NCC-induced neuronal elimination from programmed cell death in both developmental and neurodegenerative contexts. The integration of two-photon calcium imaging and genetically encoded cell-death indicators with emerging techniques such as spatially-resolved single-cell transcriptomic profiling in 3D brain tissue blocks would unravel the relationship between neuronal activity and cell death [142–144], while suggesting the molecular mechanisms that regulate neuronal activity and competition in the postnatal developing brain. Besides activity-dependent trophic factor signaling, it is unclear whether diffusible ligands and astrocytes mediate the crosstalk between neighboring neurons in a competitive niche. High-resolution secretome analysis in competing neurons will be critical for elucidating potentially novel mechanisms of neuronal competition. At the synaptic level, competition does not simply follow Hebb's rule, in which the input activity persistently strengthens or weakens the synaptic weight via a positive feedback loop, resulting in synaptic weights that are either potentiated to their maximal value or depressed to zero [89]. Heterosynaptic plasticity, which operates to inhibit runaway synaptic dynamics, has been proposed to normalize all synaptic weights within a neuron, modulating the activity of both inactive and overactivated synapses [79,90,91]. This process undoubtedly fosters benign competition between synapses and enhances the stability of the neuronal network in learning systems. However, the signals that are travelling across synapses to normalize synaptic weights and mediate synaptic competition have yet to be identified.

Finally, although the concept of selective neuronal vulnerability has emerged as being important, it remains controversial whether NCC contributes to the slowing-down of aging and neurodegenerative disorders. In hostile environments, selected populations of neurons are more vulnerable to death, whereas others exhibit resilience [3]. Cell competition has recently emerged as a mechanism for neuronal selection, offering a potential explanation for divergent cell fates [115,116]. Notably, senescent neurons in aging or degenerative brains are either eliminated by neuronal selection or retained to amplify inflammatory cascades [145]. The elimination and retention of these cells present a delicate trade-off in maintaining tissue homeostasis during aging. It is essential to determine the dynamic ratio of the elimination and retention of senescent neurons across aging and disease progression, and to elucidate the signaling pathways that mediate the crosstalk between fit and unfit neurons. Furthermore, we need to identify the inflection point at which the balance shifts and to uncover the molecular determinants of competitiveness in an aged environment, along with their interactions with senescence-inducing signals. Estimates of aging clocks and senescence entropy by using single-cell analysis [146,147], combined with cell-death indicators, will uncover the senescent state of individual neurons, the variability of senescence in a neuronal population and the extent of neuronal elimination. A comprehensive theoretical framework is needed to integrate these parameters and concepts. Promisingly, studies in neurodegenerative fly models have shown that overexpression of azot promotes cell competition and slows down Alzheimer's disease progression [12,116]. Building on this, therapeutic strategies that are aimed at modulating cell competition could offer a novel approach to combating neurodegeneration and preserving cognitive integrity.

## CONCLUDING REMARKS

While the concept of trophic factor- and activity-dependent competition between neurons has long been established, recent advances have revealed the pervasive role of cell competition across diverse spatio-temporal scales in the neural lineage. From the competition between neural progenitors that precedes neurogenesis to the interactions between various glial cell types during gliogenesis, the dynamics of cellular competition span the entire lifespan of neural cells. The consequences of NCC manifest not only in cellular viability and number, but also in the engulfment of loser stem cells, the strength of neuronal connections, the organization of dendritic arborization, the extent of astrocytic processes and the degree of territorial colonization in microglia. As complex surveillance machinery, NCC serves a multifaceted functional role: facilitating neural tube closure, optimizing the stem cell pool for brain size regulation, sculpting neural wiring, strengthening synaptic formation and modifying the spatial organization of glial cells. While target-derived trophic factors, electrical activity and intrinsic fitness fingerprints provide the general mechanisms that govern NCC, little is known about the precise signaling pathways, genetic regulators and cellular processes that trigger NCC. Moreover, distinguishing between NCC-induced cell death and physiologically regulated apoptosis in various neurological contexts, as well as determining the specific factors that establish the winner/loser status among competing neural cells, remain significant challenges.

As cellular fitness declines with age, NCC continues to act as a quality-control mechanism to eliminate unfit cells, potentially mitigating the progression of age-related neurodegeneration. In the contexts of neurodegenerative diseases and brain tumors, cell competition becomes dysregulated, leading to the accumulation of unfit neurons or the dominance of aggressive cancer cell clones. Harnessing the mechanisms of cell competition holds promise as a future therapeutic strategy to address the challenges that are posed by neural regeneration, neurodegenerative disorders and brain tumors.
